# Hsp90 Heterocomplexes Regulate Steroid Hormone Receptors: From Stress Response to Psychiatric Disease

**DOI:** 10.3390/ijms20010079

**Published:** 2018-12-25

**Authors:** Jeremy D. Baker, Ilayda Ozsan, Santiago Rodriguez Ospina, Danielle Gulick, Laura J. Blair

**Affiliations:** USF Health Byrd Institute, Morsani College of Medicine, Department of Molecular Medicine, University of South Florida, 4001 East Fowler Ave, Tampa, FL 33613, USA; jeremyb@health.usf.edu (J.D.B.); iozsan@health.usf.edu (I.O.); santiago3@health.usf.edu (S.R.O.); dgulick@health.usf.edu (D.G.)

**Keywords:** Hsp90, GR, stress response, steroid hormones, molecular chaperones, psychiatric disease, circadian rhythms, FKBP51, FKBP52, CyP40, PP5

## Abstract

The hypothalamus-pituitary-adrenal (HPA) axis directly controls the stress response. Dysregulation of this neuroendocrine system is a common feature among psychiatric disorders. Steroid hormone receptors, like glucocorticoid receptor (GR), function as transcription factors of a diverse set of genes upon activation. This activity is regulated by molecular chaperone heterocomplexes. Much is known about the structure and function of these GR/heterocomplexes. There is strong evidence suggesting altered regulation of steroid receptor hormones by chaperones, particularly the 51 kDa FK506-binding protein (FKBP51), may work with environmental factors to increase susceptibility to various psychiatric illnesses including post-traumatic stress disorder (PTSD), major depressive disorder (MDD), and anxiety. This review highlights the regulation of steroid receptor dynamics by the 90kDa heat shock protein (Hsp90)/cochaperone heterocomplexes with an in depth look at how the structural regulation and imbalances in cochaperones can cause functional effects on GR activity. Links between the stress response and circadian systems and the development of novel chaperone-targeting therapeutics are also discussed.

## 1. Introduction

**Figure 1:**
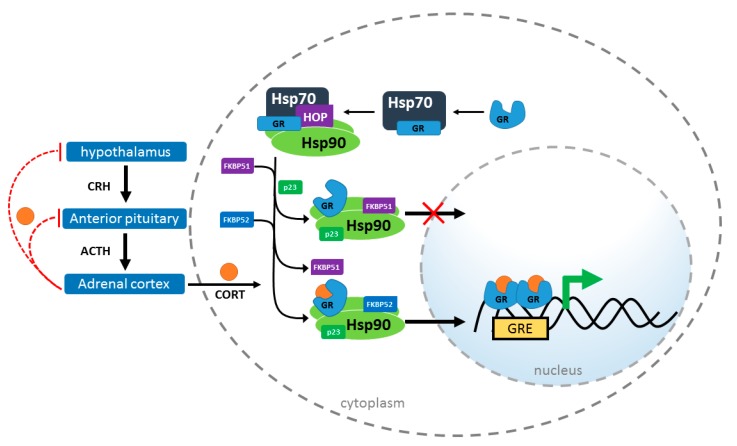
Schematic of glucocorticoid receptor (GR) transactivation in response to cortisol (CORT). After stress, the hypothalamus releases corticotropin releasing hormone (CRH) stimulating the anterior pituitary to release adrenocorticotropic hormone (ACTH). ACTH stimulates CORT release from the adrenal cortex which crosses the plasma membrane of the cell. Through negative feedback, CORT inhibits hormone release from the hypothalamus and anterior pituitary glands. Inside the cell, Hsp70 binds to and unfolds GR in the cytosol. HOP recruits GR:Hsp70 to Hsp90. Cochaperones (including FKBP51 and FKBP52) bind to Hsp90 as HOP is released. p23 binds and stabilizes the GR:Hsp90 heterocomplex. FKBP51 inhibits nuclear transactivation of GR, while FKBP52 and other copchaperones may promote translocation. Subsequently, GR binds CORT, dimerizes and translocates to the nucleus binding to glucocorticoid response elements (GREs).

The HPA, or hypothalamic-pituitary-adrenal, axis is a critical neuroendocrine system controlling numerous processes including autonomic functions (e.g., digestion), the immune response, metabolic activity, and, importantly, the stress response [1,2,3,4]. The axis consists of three major glands, for which it is named: the hypothalamus, the pituitary, and the adrenal or suprarenal gland. When stressors are encountered, including both physical insult and psychological stress, the HPA axis is activated [5,6,7]. The paraventricular nucleus of the hypothalamus releases corticotropin releasing hormone (CRH), which activates the anterior lobe of the pituitary gland causing the release of adrenocorticotropic hormone (ACTH). ACTH production stimulates the release of the glucocorticoid hormone, cortisol (CORT), from the zona fasciculata of the adrenal cortex. Then, circulating CORT inhibits the release of CRH and ACTH through a negative feedback loop ending the HPA activated stress response (Figure 1) [8,9,10]. The stress response is adaptive, but dysregulation can occur after long-term stress or other insults that can result imbalanced serum CORT [11]. 

CORT has broad physiological impacts and modulates behavior, memory, cognition, metabolism, development, inflammation, gluconeogenesis, and circadian rhythmicity [12,13]. Here, we will focus on the regulation of CORT by chaperone heterocomplexes and discuss how this relates to disruption of the normal physiological stress response. It has been well-described that short-term memory formation is intimately tied to the actions of CORT and epinephrine, particularly in response to traumatic emotional events. In the brain, CORT acts directly on the amygdala, an emotional hub, and regulates neural connections to the hippocampus, which is required for memory formation [14,15,16]. Dysregulation of CORT levels, whether positive or negative, can impair memory consolidation [17]. Memory retrieval can also be negatively impacted by the levels of CORT [18]. 

Importantly, altered CORT levels have been linked to psychiatric disorders including major depressive disorder (MDD) [19,20], general anxiety [21,22], bipolar disorder [23,24], and post-traumatic stress disorder (PTSD) [14] as well as substance use disorder [25]. CORT influences brain activity by binding glucocorticoid receptor (GR) and, to a lesser affinity, mineralocorticoid receptor (MR). Upon activation, GR homodimerizes and translocates to the nucleus where it regulates the transcription of GR-responsive genes [26]. 

GR translocation is tightly regulated by a well-characterized chaperone ensemble [27,28,29]. At the center of this complex, the 90 kDa heat shock protein (Hsp90) collaborates with cochaperones, including two FK506-binding proteins, FKBP51 and FKBP52, cyclophilin 40 (CyP40), and protein phosphatase 5 (PP5) to control GR transactivation, affecting both sensitivity to CORT and nuclear translocation [30,31]. Notably, FKBP51 affects GR transactivation in a dissimilar manner to the other cochaperones. Increased FKBP51 slows GR nuclear translocation, at least in part, through impairing the interaction between GR heterocomplexes and dynein, resulting in reduced GR activity. FKBP52, as well as CyP40 and PP5, have been shown to promote GR activity, which may be a combination of increased dynein binding as well as through displacing the inhibitory effects of FKBP51 from the Hsp90-heterocomplex, since these cochaperones bind Hsp90 at the same site (Figure 1) [32,33,34,35,36,37]. This review discusses chaperone involvement in GR physiology and the impact of chaperone imbalances that may lower resilience against psychiatric disorders. 

## 2. Chaperones in GR Signaling

The cellular stress response is highly conserved within eukaryotes [38,39], granting the ability to rapidly cope with adverse physiological insults. This process, controlled by molecular chaperones, is integral to maintaining homeostasis in all cells. A key stress response chaperone, Hsp90, is highly abundant throughout mammalian cells and uses ATP to interact with numerous substrates [40,41]. Hsp90 interacts with up to 10% of all proteins and is involved in nearly every cellular homeostatic process and is essential for signaling pathways, including GR activation [42,43,44,45,46]. Hsp90 functions as a homodimer [47]. Hsp90 consists of an ATP-binding domain at the amino-terminus separated by a flexible linker from a middle domain, which is important for client binding. Conformation of Hsp90 determines activity and function as well as regulates substrate binding [48]. Hsp90 adopts an open conformation in the absence of ATP and proceeds to a closed state when it is ATP-bound [49]. In addition, cytosolic Hsp90 contains a carboxy-terminal MEEVD (met-glu-glu-val-asp) motif that regulates the interaction with a host of cochaperones [50]. This allows for direct binding of cochaperones containing a tetratricopeptide repeat (TPR) domain, including FKBP51, FKBP52, and CyP40. 

Hsp90 is regulated transcriptionally by heat shock factor 1 (HSF1), posttranslationally by modifications including phosphorylation and acetylation [50], and functionally by a diverse set of cochaperones [51]. Although the main binding of FKBP51, FKBP52, CyP40, and PP5 is through a conserved TPR domain, cochaperones have been shown to bind to Hsp90 in all three domains [50]. These cochaperones compete for Hsp90 binding; however, simultaneous binding of more than one cochaperone has also been demonstrated [52,53]. Cochaperones influence Hsp90 substrate recognition, alter Hsp90 ATPase activity and conformational dynamics, and have the ability to interact with Hsp90 substrates directly [54,55]. 

### Hsp90 Heterocomplex

It has been demonstrated, in vitro, that an Hsp90 heterocomplex is required for GR maturation [31,56]. In a stepwise process, GR interacts first with Hsp70, and then is passed to Hsp90 via Hsp70-Hsp90 organizing protein (HOP) [46,57,58]. HOP is dislodged from this Hsp90-GR complex upon Hsp90 binding ATP and subsequent association of cochaperones, FKBP51, FKBP52, CyP40, or PP5 [59,60]. p23 preferentially associates with ATP-bound Hsp90 through the N-terminus and middle domains stabilizing the complex in a conformation with high affinity for CORT [30,61]. Upon CORT binding, GR dimerizes and translocates into the nucleus where it regulates the transcription of GR-responsive genes. 

With the development of new technologies to evaluate the detailed structure of large, multiprotein complexes, we are continually learning more about how these proteins interact. This information is important to understand how changes in structure are tightly linked to functional effects. Recently, cryoelectron microscopy has further clarified the interactions between Hsp70 and Hsp90 that are required for GR maturation and function [46]. It was shown that the coupling of the ATP-dependent chaperone cycles of Hsp70 and Hsp90 may be required for GR maturation. Using recombinant proteins and the ligand binding domain of GR (GRLBD), it was shown that GRLBD is first unfolded by Hsp70 and inactivated. Hsp90 reverses this step resulting in folded, aggregation resistant, and functional GRLBD. This could explain the necessity of Hsp90 in GR maturation. 

Remarkably, during the hand-off of GR from Hsp70 to Hsp90, the substrate binding domains of Hsp70 and client binding domain of Hsp90 align. HOP recruits GRLBD:Hsp70 to Hsp90 resulting in an intermediary complex of GRLBD:Hsp70:Hsp90:HOP in which GRLBD is physically in contact with both Hsp70 and Hsp90 simultaneously [46]. This conformation allows for an interaction between the ATP domains of Hsp90 and Hsp70 and subsequent coupling of ATP hydrolysis. A key finding is that Hsp90 ATP hydrolysis is required for client loading from Hsp70, however blocking ATPase activity does not perturb the GRLBD:Hsp70:Hsp90:HOP intermediary complex. This is important because it helps to explain how inhibition of Hsp90 ATPase activity results in Hsp70-mediated degradation, as client loading is blocked. It was recently described that the intermediary complex actually contains two Hsp70 molecules, where one Hsp70 delivers GR to Hsp90, while the other supports the HOP interaction [62]. Overall, the Hsp70-Hsp90 system maintains GR in a competent high-affinity state for CORT, allowing for response to changing CORT levels. This provides an explanation for the necessity of this cycle for GR activity. Further studies are needed to evaluate how cochaperones affect this heterocomplex to functionally regulate GR.

Rearrangement of Hsp90 by cochaperones, in addition to ATP-mediated effects [50], directly impacts client binding [30]. For example, recent nuclear magnetic resonance spectroscopy studies have solved the structure of the Hsp90/FKBP51 [48]. FKBP51 binds between the two dimers of Hsp90 and upon binding stabilizes Hsp90 in the open conformation, reducing ATP hydrolysis activity. Other TPR-containing cochaperones may share the same Hsp90 binding site, but potentially have disparate effects on Hsp90 conformation. Furthermore, it has been found that GR preferentially binds Hsp90 when in the closed ATP-bound state, where both a TPR-containing protein and HOP are associated [30]. Given this, Hsp90/FKBP51 association may disrupt Hsp90/GR binding.

The relationship of cochaperones to CORT levels is complex as cochaperones may have diverse effects on GR and CORT physiology. FKBP51 is upregulated by GR activity, which directly promotes the transcription of *FKBP5*, the gene that encodes FKBP51 [63]. FKBP51, then, negatively inhibits GR activity. The proposed mechanism of action is a short, negative feedback loop whereby FKBP51, in an Hsp90-dependent mechanism, decreases the binding of CORT to GR leading to CORT resistance [64]. *FKBP4*, the gene that encodes the highly similar FKBP51 homolog, FKBP52, may have an opposing effect on GR activity; however, conflicting evidence suggests FKBP52 may not alter GR nuclear transactivation [32,35,36,65,66]. Another TPR-containing cochaperone, CyP40, can also regulate GR through an Hsp90 heterocomplex. It has also been reported that CyP40 may facilitate the export of CORT from the nucleus [65]. CyP40, like FKBP52 and PP5, interacts with dynein, which is a cytoskeletal motor protein that can regulate GR transport [67]. Evidence suggests that PP5 binds to the Hsp90/GR complex at an intermediate step in CORT activation, following the binding of FKBP51 during the basal state [68,69]. PP5 has also been shown to dephosphorylate GR, which can alter GR activity [70]. Thus, PP5 may regulate GR activity through two distinct, but linked mechanisms.

Interestingly, it has been shown that about half of GR within the cell is in complex with Hsp90 and FKBP51 or FKBP52 [68]. About one third of GR is in complex with Hsp90 and PP5, and only a fraction of GR has been found in an Hsp90/CyP40 complex. However, since cochaperones compete to bind Hsp90, this normal distribution of GR/Hsp90 heterocomplexes may become imbalanced with alterations of any TPR-containing proteins, potentially disrupting GR regulation and the stress response.

## 3. Chaperones Implicated in Psychiatric Disorders

Cochaperone variants and altered expression levels have been linked to psychiatric illness [71,72]. Moreover, cochaperone dysregulation may also contribute to circadian desynchrony, which is exacerbated by and implicated in the etiology of mood disorders. Candidate studies have identified common single nucleotide polymorphisms (SNPs) in the gene that encodes FKBP51, *FKBP5*, that interact synergistically with environmental factors to increase susceptibility to develop PTSD, MDD, anxiety, and bipolar disorder [73,74,75]. Some of these *FKBP5* SNPs result in increased FKBP51 levels following stress [76]. Increased FKBP51 reduces GR sensitivity, prolonging the HPA-mediated stress response and resulting in increased circulating CORT [77]. We and others have shown that mice lacking *Fkbp5* (*Fkbp5*^−/−^ mice) are resilient to depressive-like behavior following stress [78,79]. These mice have reduced levels of serum CORT following restraint stress or exogenous CORT administration, suggesting that these stress-resilient phenotypes may be linked to CORT regulation by FKBP51. 

FKBP52, CyP40 and PP5 have not been currently linked to psychiatric disease, but since each of these cochaperones play an important role in GR regulation through Hsp90, it is possible that dysregulation could increase risk. Work in vitro and in vivo has started to reveal the physiological implications of imbalances in these cochaperones. A recent study in mice with reduced levels of FKBP52 revealed that FKBP52 may not have an apparent role in regulating anxiety-like behaviors or recognition memory, including fear conditioning, but rather may be more important in motor coordination [80]. This corroborated previous work that did not detect any GR-related physiological changes in FKBP52 knockout mice [66]. However, since FKBP52 competes with FKBP51 to bind Hsp90, it has been suggested that reduced FKBP52 levels could regulate the stress response by increasing the Hsp90/FKBP51 interaction [63]. Recent work identified a role for CyP40 in the regulation of amygdala-mediated fear extinction [81]. An NIH-led study identified CyP40 as being enriched in the basolateral amygdala of normal mice, however, CyP40 expression is reduced in mice that demonstrate impairments in extinction learning. This group also showed that CyP40 colocalizes with GR in these mice and that the extinction regulating effects can be blocked by a GR antagonist. This work may provide a role for CyP40 in PTSD. A specific role in stress-related phenotypes has been not yet been described for PP5. However, GR-specific effects may be difficult to identify, since PP5 primarily functions as a protein phosphatase.

HPA hyperactivity has been extensively shown for depressed individuals. GR dysfunction contributes to this hyperactivity [82,83,84]. There is evidence that the use of antidepressants inhibits transcription of genes with glucocorticoid responsive elements (GREs). Further, antidepressants increase GR expression in patients. It has been shown that antidepressants have activity beyond targeting monoamine transporters, since they also upregulate autophagy markers [85]. FKBP51 enhances the activity of some antidepressants, which may be a result of priming autophagy pathways. Further, FKBP51 inhibition improved stress coping behavior in a mouse model of depression co-treated with a selective serotonin reuptake inhibitor (SSRI), while decreasing anti-anxiety effects of the SSRI [86]. In further support of a role for FKBP51 in modulating antidepressant effects, it has been shown that *FKBP5* SNPs affect antidepressant response [87,88]. It should also be noted however, that a separate study in geriatric depressed patients showed no role for *FKBP5* SNPs in antidepressant efficacy prediction [89]. Additionally, it has been shown that depression may be linked to epigenetic changes through methylation or differential gene expression. Some antidepressants may mediate epigenetic changes; however, FKBP51 may affect this activity. High FKBP51 expression reduces the activity of DNA methyltransferase, DNMT1, by modulating its activating kinases. This reduction in DNMT1 activity reduces DNA methylation, which broadly impacts expression of stress-induced genes and alters antidepressant activity [90]. 

## 4. Stress Response and Circadian Rhythmicity

**Figure 2:**
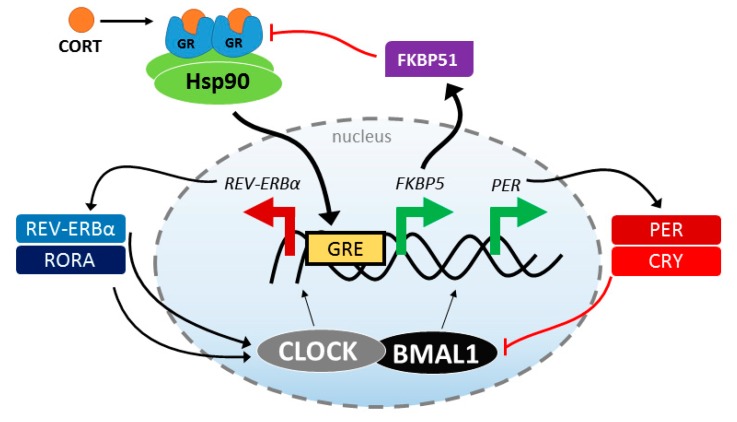
Schematic of the feedback between the molecular clock and stress response systems. Stress produces CORT, which binds to the GR/Hsp90 heterocomplex. GR forms a homodimer and translocate to the nucleus where it binds the glucocorticoid response elements in the promoter region. This leads to increased FKBP5/FKBP51, which slows GR activity, and increased PER expression, a component of the negative arm of the circadian clock; at the same time, REV-ERBα, a positive arm protein is down regulated.

Physiological stress is frequently associated with circadian disruption, and the regulation of the stress response system is one mechanism by which circadian rhythms are altered [91]. Thus, it is not surprising that disruptions in the circadian rhythms are a common symptom across stress-related psychiatric disorders [92]. Similar to the stress response system, the circadian clock is a well-conserved mechanism that allows organisms to adapt to their environment. This homeostasis occurs not only in individual tissues, but in the coordination within and between systems. Interestingly, there are multiple interactions between the HPA axis and the circadian system, including circadian control over the daily cycling of CORT release [93] and stress reactivity [94]. There is also a growing body of literature suggesting that CORT synchronizes the rhythms of peripheral clocks [95], and even modulates that rhythms of central nervous system clocks outside of the mast oscillator in the suprachiasmatic nucleus [96]. Furthermore, it is likely that GR activity is an important link between these two systems. As already described, CORT promotes GR activation, transcriptionally regulating many genes that contain GREs. This includes core clock genes, *PERIOD 1* and *PERIOD 2*, and the accessory clock genes, *REV-ERBα* and *RORA*. The latter genes are essential for the normal activity of the positive arm of the clock via BMAL1 transcription factor activity [97]. Knockdown of either the positive (BMAL1) or negative (PERIOD genes) arm of the clock disrupts circadian rhythmicity at both the molecular and physiological levels [98,99,100]. This suggests that the regulation of GR by the Hsp90 heterocomplex can directly impact the expression of clock genes and circadian rhythmicity at a cellular and organismal level, as summarized in Figure 2. This is particularly interesting for FKBP51, since the *FKBP5* gene also contains GREs [101], so the levels of FKBP51 are increased by GR activation. Since FKBP51 works in a short, negative feedback loop with GR, increased FKBP51 levels may directly regulate the activation of the negative feedback loop of the clock FKBP51 levels can be affected not only by SNPs, but they also dramatically increase with age [102,103]. Sleep architecture and quality decline with age, largely due to impairments in circadian rhythmicity [104]. Thus, strategies aimed at restoring CORT homeostasis or depleting FKBP51 could be beneficial for both acute and chronic stress-related co-morbidities, like circadian rhythm sleep disorders. In support of this, mice lacking *Fkbp5* demonstrated increased wake times and protection from stress-induced sleep disruption [105]. More work needs to be done to fully understand the connection between these essential processes, but it is possible that finding treatments that restore normal stress response or circadian rhythmicity may be beneficial for both systems. 

## 5. Therapeutic Progress

It is clear that selective inhibitory molecules are not only needed for therapeutic interventions, but also as tools to investigate the complex interplay at work in diverse chaperone heterocomplexes. Although Hsp90 inhibitors are available, because of the vast network of Hsp90-interactions, numerous on- and off-target effects are a primary concern [106]. Geldanamycin (GA), a long-used Hsp90 inhibitor, causes cytotoxicity through the production of reactive oxygen species, which can result in hepato- and ocular toxicity [107,108,109]. Additionally, the GA backbone binds to ion channels of the mitochondrial membrane resulting in increased Ca^2+^ levels [110]. Furthermore, because the N-terminal of Hsp90 includes a conserved fold for binding ATP, inhibitors targeting this region can inadvertently inhibit other important ATP-binding proteins. For example, radicicol has been shown to inhibit the activity of a Type II DNA topoisomerase [111]. In addition, N-terminal Hsp90 ATP inhibitors activate the stress response, which upregulates Hsp70, Hsp40, and Hsp27 along with other pro-survival factors [112]. With this in mind, targeting Hsp90 cochaperones may be a promising alternative therapeutic approach. 

The principal tool in determining FKBP51 and FKBP52 functional activity has been FK506 (also tacrolimus), a potent immunosuppressant. FK506 binds FKBP51 and FKBP52, disrupting signaling events mediated by the calcium-dependent serine/threonine protein phosphatase, calcineurin (CaN/PP2B). FK506 will non-specifically bind to FKBPs. Therefore, specific inhibitors that work through an alternate mechanism are needed to discriminate between the FKBPs, especially the highly homologous FKBP51 and FKBP52. 

Using induced-fit modeling, highly selective inhibitors of FKBP51, SAFit 1 and SAFit2, have now been generated [113]. SAFit1 showed protection from FKBP51-mediated neurite outgrowth suppression in primary neurons, while SAFit2 treatment in mice led to antidepressant-like effects [113]. Additionally, microRNA-511 (miR-511), a non-coding RNA molecule, was shown to silence *FKBP5* post-transcriptionally [114]. miR-511 suppressed CORT-induced upregulation of FKBP51 and promote neurite outgrowth in primary neurons. miR-511 may be a promising therapeutic candidate for suppressing FKBP51. Recently, benztropine was shown to restore GR activity in the presence of high FKBP51 and interact with FKBP51, but not FKBP52 [77]. 

Selective inhibitors for FKBP52, CyP40, and PP5 have not been reported. Structural insights may help guide strategies to target these chaperones. For example, the proline-rich loop extending over the FK1 catalytic domain of FKBP52 has been described and may be targetable, since this domain is suggested to have a role GR regulation [115]. However, inhibiting FKBP52 may lead to reduced fertility, as this has been found in mice lacking this protein [116]. Additional studies are still needed to better understand the regions on each cochaperone that are most important for regulating GR activity. At the same time, further development of selective inhibitors will both benefit from and aid these studies.

## 6. Conclusions

Although there has been substantial research investigating the role of GR in regulating the stress response, recent work has advanced our understanding of both the structural and functional regulation of GR by Hsp90 heterocomplexes. There is a growing body of evidence describing the functional effects of cochaperones on GR activity. Still there is much to learn about how these cochaperones regulate GR signaling in vivo and how the intracellular feedback loops and HPA axis are interconnected. Even less well known are the structural effects of cochaperones on GR-Hsp90 heterocomplexes. More work needs to be done to elucidate the effects of cochaperones on GR structure, function, and feedback regulation.

The stress response has now been suggested to be linked to circadian rhythms. However, a detailed investigation at the molecular level has yet to be done, despite apparent overlaps in regulation through GR activity. Additional studies are needed to start to understand how stress response and circadian rhythms are molecularly linked and how this link impacts the susceptibility and severity of psychiatric disorders.

## Abbreviations

ACTH	adrenocorticotropic hormone
ATP	adenosine triphosphate
CORT	cortisol
CRH	corticotropin-releasing hormone
CaN/PP2B	calcineurin
CyP40	cyclophilin 40
FKBP	FK506-binding protein
GR	glucocorticoid receptor
GRE	glucocorticoid response element
HPA	hypothalamic-pituitary-adrenal
Hsp90	heat shock protein 90
LBD	ligand-binding domain
MDD	major depressive disorder
MEEVD	binding motif in Hsp90 that binds TPR domain
MR	mineralocorticoid receptors
N2A	Neuro2A, a mouse neuroblastoma cell line
PTSD	post-traumatic stress disorder
SNP	single nucleotide polymorphism
TPR	tetratricopeptide repeat

## References

[1] Holsboer F. (2000). The corticosteroid receptor hypothesis of depression. Neuropsychopharmacol. Off. Publ. Am. Coll. Neuropsychopharmacol..

[2] Eisenlohr-Moul T.A., Miller A.B., Giletta M., Hastings P.D., Rudolph K.D., Nock M.K., Prinstein M.J. (2018). HPA axis response and psychosocial stress as interactive predictors of suicidal ideation and behavior in adolescent females: A multilevel diathesis-stress framework. Neuropsychopharmacol. Off. Publ. Am. Coll. Neuropsychopharmacol..

[3] Daskalakis N.P., Lehrner A., Yehuda R. (2013). Endocrine Aspects of Post-traumatic Stress Disorder and Implications for Diagnosis and Treatment. Endocrinol. Metab. Clin. N. Am..

[4] McEwen B.S. (1998). Protective and damaging effects of stress mediators. N. Engl. J. Med..

[5] Stephens M.A.C., Wand G. (2012). Stress and the HPA axis: Role of glucocorticoids in alcohol dependence. Alcohol. Res. Curr. Rev..

[6] Rose A.K., Shaw S.G., Prendergast M.A., Little H.J. (2010). The importance of glucocorticoids in alcohol dependence and neurotoxicity. Alcohol. Clin. Exp. Res..

[7] Smith S.M., Vale W.W. (2006). The role of the hypothalamic-pituitary-adrenal axis in neuroendocrine responses to stress. Dialogues Clin. Neurosci..

[8] Keller-Wood M. (2015). Hypothalamic-Pituitary—Adrenal Axis-Feedback Control. Compr. Physiol..

[9] Zhe D., Fang H., Yuxiu S. (2008). Expressions of hippocampal mineralocorticoid receptor (MR) and glucocorticoid receptor (GR) in the single-prolonged stress-rats. Acta Histochem. Cytochem..

[10] Herman J.P., Patel P.D., Akil H., Watson S.J. (1989). Localization and regulation of glucocorticoid and mineralocorticoid receptor messenger RNAs in the hippocampal formation of the rat. Mol. Endocrinol. (Baltimore MD.).

[11] Qin D.-D., Rizak J., Feng X.-L., Yang S.-C., Lü L.-B., Pan L., Yin Y., Hu X.-T. (2016). Prolonged secretion of cortisol as a possible mechanism underlying stress and depressive behaviour. Sci. Rep..

[12] Hannibal K.E., Bishop M.D. (2014). Chronic stress, cortisol dysfunction, and pain: A psychoneuroendocrine rationale for stress management in pain rehabilitation. Phys. Ther..

[13] Fries G.R., Vasconcelos-Moreno M.P., Gubert C., dos Santos B.T.M.Q., Sartori J., Eisele B., Ferrari P., Fijtman A., Rüegg J., Gassen N.C. (2014). Hypothalamic-pituitary-adrenal axis dysfunction and illness progression in bipolar disorder. Int. J. Neuropsychopharmacol..

[14] Meewisse M.L., Reitsma J.B., de Vries G.J., Gersons B.P., Olff M. (2007). Cortisol and post-traumatic stress disorder in adults: Systematic review and meta-analysis. Br. J. Psychiatry J. Ment. Sci..

[15] Tatomir A., Micu C., Crivii C. (2014). The impact of stress and glucocorticoids on memory. Clujul Med. (1957).

[16] Roozendaal B., McEwen B.S., Chattarji S. (2009). Stress, memory and the amygdala. Nat. Rev. Neurosci..

[17] de Quervain D.J., Roozendaal B., McGaugh J.L. (1998). Stress and glucocorticoids impair retrieval of long-term spatial memory. Nature.

[18] Wolf O.T., Kuhlmann S., Buss C., Hellhammer D.H., Kirschbaum C. (2004). Cortisol and memory retrieval in humans: Influence of emotional valence. Ann. N. Y. Acad. Sci..

[19] Dedovic K., Ngiam J. (2015). The cortisol awakening response and major depression: Examining the evidence. Neuropsychiatr. Dis. Treat..

[20] Dougherty L.R., Klein D.N., Olino T.M., Dyson M., Rose S. (2009). Increased waking salivary cortisol and depression risk in preschoolers: The role of maternal history of melancholic depression and early child temperament. J. Child Psychol. Psychiatry Allied Discip..

[21] Otte C., Hart S., Neylan T.C., Marmar C.R., Yaffe K., Mohr D.C. (2005). A meta-analysis of cortisol response to challenge in human aging: Importance of gender. Psychoneuroendocrinology.

[22] Chaudieu I., Beluche I., Norton J., Boulenger J.P., Ritchie K., Ancelin M.L. (2008). Abnormal reactions to environmental stress in elderly persons with anxiety disorders: Evidence from a population study of diurnal cortisol changes. J. Affect. Disord..

[23] Cervantes P., Gelber S., Kin F.N., Nair V.N., Schwartz G. (2001). Circadian secretion of cortisol in bipolar disorder. J. Psychiatry Neurosci. JPN.

[24] Joyce P.R., Donald R.A., Elder P.A. (1987). Individual differences in plasma cortisol changes during mania and depression. J. Affect. Disord..

[25] Wand G. (2008). The influence of stress on the transition from drug use to addiction. Alcohol. Res. Health J. Natl. Inst. Alcohol Abuse Alcohol..

[26] Meijsing S.H. (2015). Mechanisms of Glucocorticoid-Regulated Gene Transcription. Adv. Exp. Med. Biol..

[27] Echeverría P.C., Mazaira G., Erlejman A., Gomez-Sanchez C., Piwien Pilipuk G., Galigniana M.D. (2009). Nuclear import of the glucocorticoid receptor-hsp90 complex through the nuclear pore complex is mediated by its interaction with Nup62 and importin beta. Mol. Cell. Biol..

[28] Pratt W.B., Galigniana M.D., Morishima Y., Murphy P.J. (2004). Role of molecular chaperones in steroid receptor action. Essays Biochem..

[29] Noguchi T., Makino S., Matsumoto R., Nakayama S., Nishiyama M., Terada Y., Hashimoto K. (2010). Regulation of glucocorticoid receptor transcription and nuclear translocation during single and repeated immobilization stress. Endocrinology.

[30] Lorenz O.R., Freiburger L., Rutz D.A., Krause M., Zierer B.K., Alvira S., Cuellar J., Valpuesta J.M., Madl T., Sattler M. (2014). Modulation of the Hsp90 chaperone cycle by a stringent client protein. Mol. Cell.

[31] Picard D., Khursheed B., Garabedian M.J., Fortin M.G., Lindquist S., Yamamoto K.R. (1990). Reduced levels of hsp90 compromise steroid receptor action in vivo. Nature.

[32] Wochnik G.M., Ruegg J., Abel G.A., Schmidt U., Holsboer F., Rein T. (2005). FK506-binding proteins 51 and 52 differentially regulate dynein interaction and nuclear translocation of the glucocorticoid receptor in mammalian cells. J. Biol. Chem..

[33] Galigniana M.D., Radanyi C., Renoir J.M., Housley P.R., Pratt W.B. (2001). Evidence that the peptidylprolyl isomerase domain of the hsp90-binding immunophilin FKBP52 is involved in both dynein interaction and glucocorticoid receptor movement to the nucleus. J. Biol. Chem..

[34] Storer C.L., Dickey C.A., Galigniana M.D., Rein T., Cox M.B. (2011). FKBP51 and FKBP52 in signaling and disease. Trends Endocrinol. Metab. TEM.

[35] Riggs D.L., Roberts P.J., Chirillo S.C., Cheung-Flynn J., Prapapanich V., Ratajczak T., Gaber R., Picard D., Smith D.F. (2003). The Hsp90-binding peptidylprolyl isomerase FKBP52 potentiates glucocorticoid signaling in vivo. EMBO J..

[36] Riggs D.L., Cox M.B., Tardif H.L., Hessling M., Buchner J., Smith D.F. (2007). Noncatalytic role of the FKBP52 peptidyl-prolyl isomerase domain in the regulation of steroid hormone signaling. Mol. Cell. Biol..

[37] Banerjee A., Periyasamy S., Wolf I.M., Hinds T.D., Yong W., Shou W., Sanchez E.R. (2008). Control of glucocorticoid and progesterone receptor subcellular localization by the ligand-binding domain is mediated by distinct interactions with tetratricopeptide repeat proteins. Biochemistry.

[38] Johnson J.L. (2012). Evolution and function of diverse Hsp90 homologs and cochaperone proteins. Biochim. Biophys. Acta (BBA) Mol. Cell Res..

[39] Liu X.D., Liu P.C., Santoro N., Thiele D.J. (1997). Conservation of a stress response: Human heat shock transcription factors functionally substitute for yeast HSF. EMBO J..

[40] Picard D. (2002). Heat-shock protein 90, a chaperone for folding and regulation. Cell. Mol. Life Sci. CMLS.

[41] Zhao R., Davey M., Hsu Y.-C., Kaplanek P., Tong A., Parsons A.B., Krogan N., Cagney G., Mai D., Greenblatt J. (2005). Navigating the Chaperone Network: An Integrative Map of Physical and Genetic Interactions Mediated by the Hsp90 Chaperone. Cell.

[42] Wu Z., Gholami A.M., Kuster B. (2012). Systematic identification of the HSP90 candidate regulated proteome. Mol. Cell. Proteomics MCP.

[43] Mailhos C., Howard M.K., Latchman D.S. (1994). Heat shock proteins hsp90 and hsp70 protect neuronal cells from thermal stress but not from programmed cell death. J. Neurochem..

[44] Gallo L.I., Lagadari M., Piwien-Pilipuk G., Galigniana M.D. (2011). The 90-kDa heat-shock protein (Hsp90)-binding immunophilin FKBP51 is a mitochondrial protein that translocates to the nucleus to protect cells against oxidative stress. J. Biol. Chem..

[45] Swaminathan S. (2005). Protein quality control: Knowing when to fold. Nat. Cell Biol..

[46] Kirschke E., Goswami D., Southworth D., Griffin P.R., Agard D.A. (2014). Glucocorticoid Receptor Function Regulated by Coordinated Action of the Hsp90 and Hsp70 Chaperone Cycles. Cell.

[47] Li J., Buchner J. (2013). Structure, function and regulation of the hsp90 machinery. Biomed. J..

[48] Oroz J., Chang B.J., Wysoczanski P., Lee C.-T., Pérez-Lara Á., Chakraborty P., Hofele R.V., Baker J.D., Blair L.J., Biernat J., Urlaub H. (2018). Structure and pro-toxic mechanism of the human Hsp90/PPIase/Tau complex. Nat. Commun..

[49] Hellenkamp B., Wortmann P., Kandzia F., Zacharias M., Hugel T. (2017). Multidomain structure and correlated dynamics determined by self-consistent FRET networks. Nat. Methods.

[50] Schopf F.H., Biebl M.M., Buchner J. (2017). The HSP90 chaperone machinery. Nat. Rev. Mol. Cell Biol..

[51] Mayer M.P., Le Breton L. (2015). Hsp90: Breaking the symmetry. Mol. Cell.

[52] Hildenbrand Z.L., Molugu S.K., Herrera N., Ramirez C., Xiao C., Bernal R.A. (2011). Hsp90 can accommodate the simultaneous binding of the FKBP52 and HOP proteins. Oncotarget.

[53] Harst A., Lin H., Obermann W.M.J. (2005). Aha1 competes with Hop, p50 and p23 for binding to the molecular chaperone Hsp90 and contributes to kinase and hormone receptor activation. Biochem. J..

[54] Zuehlke A., Johnson J.L. (2010). Hsp90 and co-chaperones twist the functions of diverse client proteins. Biopolymers.

[55] Sahasrabudhe P., Rohrberg J., Biebl M.M., Rutz D.A., Buchner J. (2017). The Plasticity of the Hsp90 Co-chaperone System. Mol. Cell.

[56] Dittmar K.D., Hutchison K.A., Owens-Grillo J.K., Pratt W.B. (1996). Reconstitution of the steroid receptor.hsp90 heterocomplex assembly system of rabbit reticulocyte lysate. J. Biol. Chem..

[57] Röhl A., Wengler D., Madl T., Lagleder S., Tippel F., Herrmann M., Hendrix J., Richter K., Hack G., Schmid A.B. (2015). Hsp90 regulates the dynamics of its cochaperone Sti1 and the transfer of Hsp70 between modules. Nat. Commun..

[58] Chrousos G.P., Kino T. (2009). Glucocorticoid signaling in the cell. Expanding clinical implications to complex human behavioral and somatic disorders. Ann. N. Y. Acad. Sci..

[59] Grenert J.P., Johnson B.D., Toft D.O. (1999). The importance of ATP binding and hydrolysis by hsp90 in formation and function of protein heterocomplexes. J. Biol. Chem..

[60] Echeverria P.C., Picard D. (2010). Molecular chaperones, essential partners of steroid hormone receptors for activity and mobility. Biochim. Biophys. Acta.

[61] Morishima Y., Kanelakis K.C., Murphy P.J., Lowe E.R., Jenkins G.J., Osawa Y., Sunahara R.K., Pratt W.B. (2003). The hsp90 cochaperone p23 is the limiting component of the multiprotein hsp90/hsp70-based chaperone system in vivo where it acts to stabilize the client protein: Hsp90 complex. J. Biol. Chem..

[62] Blair L.J., Genest O., Mollapour M. (2018). The multiple facets of the Hsp90 machine. Nat. Struct. Mol. Biol..

[63] Tatro E.T., Everall I.P., Kaul M., Achim C.L. (2009). Modulation of glucocorticoid receptor nuclear translocation in neurons by immunophilins FKBP51 and FKBP52: Implications for major depressive disorder. Brain Res..

[64] Zannas A.S., Wiechmann T., Gassen N.C., Binder E.B. (2016). Gene-Stress-Epigenetic Regulation of FKBP5: Clinical and Translational Implications. Neuropsychopharmacol. Off. Publ. Am. Coll. Neuropsychopharmacol..

[65] Davies T.H., Ning Y.M., Sanchez E.R. (2005). Differential control of glucocorticoid receptor hormone-binding function by tetratricopeptide repeat (TPR) proteins and the immunosuppressive ligand FK506. Biochemistry.

[66] Wolf I.M., Periyasamy S., Hinds T., Yong W., Shou W., Sanchez E.R. (2009). Targeted ablation reveals a novel role of FKBP52 in gene-specific regulation of glucocorticoid receptor transcriptional activity. J. Steroid Biochem. Mol. Biol..

[67] Galigniana M.D., Harrell J.M., Murphy P.J., Chinkers M., Radanyi C., Renoir J.M., Zhang M., Pratt W.B. (2002). Binding of hsp90-associated immunophilins to cytoplasmic dynein: Direct binding and in vivo evidence that the peptidylprolyl isomerase domain is a dynein interaction domain. Biochemistry.

[68] Silverstein A.M., Galigniana M.D., Chen M.S., Owens-Grillo J.K., Chinkers M., Pratt W.B. (1997). Protein phosphatase 5 is a major component of glucocorticoid receptor.hsp90 complexes with properties of an FK506-binding immunophilin. J. Biol. Chem..

[69] Golden T., Swingle M., Honkanen R.E. (2008). The role of serine/threonine protein phosphatase type 5 (PP5) in the regulation of stress-induced signaling networks and cancer. Cancer Metastasis Rev..

[70] Wang Z., Chen W., Kono E., Dang T., Garabedian M.J. (2007). Modulation of glucocorticoid receptor phosphorylation and transcriptional activity by a C-terminal-associated protein phosphatase. Mol. Endocrinol..

[71] O’Leary J.C., Zhang B., Koren J., Blair L., Dickey C.A. (2013). The role of FKBP5 in mood disorders: Action of FKBP5 on steroid hormone receptors leads to questions about its evolutionary importance. CNS Neurol. Disord. Drug Targets.

[72] Jaaskelainen T., Makkonen H., Palvimo J.J. (2011). Steroid up-regulation of FKBP51 and its role in hormone signaling. Curr. Opin. Pharmacol..

[73] Binder E.B. (2009). The role of FKBP5, a co-chaperone of the glucocorticoid receptor in the pathogenesis and therapy of affective and anxiety disorders. Psychoneuroendocrinology.

[74] Criado-Marrero M., Rein T., Binder E.B., Porter J.T., Koren J., Blair L.J. (2018). Hsp90 and FKBP51: Complex regulators of psychiatric diseases. Philos. Trans. R. Soc. B Biol.Sci..

[75] Xie P., Kranzler H.R., Poling J., Stein M.B., Anton R.F., Farrer L.A., Gelernter J. (2010). Interaction of FKBP5 with childhood adversity on risk for post-traumatic stress disorder. Neuropsychopharmacol. Off. Publ. Am. Coll. Neuropsychopharmacol..

[76] Klengel T., Mehta D., Anacker C., Rex-Haffner M., Pruessner J.C., Pariante C.M., Pace T.W., Mercer K.B., Mayberg H.S., Bradley B. (2013). Allele-specific FKBP5 DNA demethylation mediates gene-childhood trauma interactions. Nat. Neurosci..

[77] Sabbagh J.J., Cordova R.A., Zheng D., Criado-Marrero M., Lemus A., Li P., Baker J.D., Nordhues B.A., Darling A.L., Martinez-Licha C. (2018). Targeting the FKBP51/GR/Hsp90 Complex to Identify Functionally Relevant Treatments for Depression and PTSD. ACS Chem. Biol..

[78] O’Leary J.C., Dharia S., Blair L.J., Brady S., Johnson A.G., Peters M., Cheung-Flynn J., Cox M.B., de Erausquin G., Weeber E.J. (2011). A new anti-depressive strategy for the elderly: Ablation of FKBP5/FKBP51. PLoS ONE.

[79] Touma C., Gassen N.C., Herrmann L., Cheung-Flynn J., Bull D.R., Ionescu I.A., Heinzmann J.M., Knapman A., Siebertz A., Depping A.M. (2011). FK506 binding protein 5 shapes stress responsiveness: Modulation of neuroendocrine reactivity and coping behavior. Biol. Psychiatry.

[80] Young M.J., Geiszler P.C., Pardon M.C. (2016). A novel role for the immunophilin FKBP52 in motor coordination. Behav. Brain Res..

[81] Gunduz-Cinar O., Brockway E., Lederle L., Wilcox T., Halladay L.R., Ding Y., Oh H., Busch E.F., Kaugars K., Flynn S. (2018). Identification of a novel gene regulating amygdala-mediated fear extinction. Mol. Psychiatry.

[82] Vreeburg S.A., Hoogendijk W.G., van Pelt J., Derijk R.H., Verhagen J.C., van Dyck R., Smit J.H., Zitman F.G., Penninx B.W. (2009). Major depressive disorder and hypothalamic-pituitary-adrenal axis activity: Results from a large cohort study. Arch. Gen. Psychiatry.

[83] Varghese F.P., Brown E.S. (2001). The Hypothalamic-Pituitary-Adrenal Axis in Major Depressive Disorder: A Brief Primer for Primary Care Physicians. Primary Care Companion J. Clin. Psychiatry.

[84] Pariante C.M., Lightman S.L. (2008). The HPA axis in major depression: Classical theories and new developments. Trends Neurosci..

[85] Gassen N.C., Hartmann J., Zschocke J., Stepan J., Hafner K., Zellner A., Kirmeier T., Kollmannsberger L., Wagner K.V., Dedic N. (2014). Association of FKBP51 with priming of autophagy pathways and mediation of antidepressant treatment response: Evidence in cells, mice, and humans. PLoS Med..

[86] Pöhlmann M.L., Häusl A.S., Harbich D., Balsevich G., Engelhardt C., Feng X., Breitsamer M., Hausch F., Winter G., Schmidt M.V. (2018). Pharmacological Modulation of the Psychiatric Risk Factor FKBP51 Alters Efficiency of Common Antidepressant Drugs. Front. Behav. Neurosci..

[87] Binder E.B., Salyakina D., Lichtner P., Wochnik G.M., Ising M., Pütz B., Papiol S., Seaman S., Lucae S., Kohli M.A. (2004). Polymorphisms in FKBP5 are associated with increased recurrence of depressive episodes and rapid response to antidepressant treatment. Nat. Genet..

[88] Lekman M., Laje G., Charney D., Rush A.J., Wilson A.F., Sorant A.J.M., Lipsky R., Wisniewski S.R., Manji H., McMahon F.J. (2008). The FKBP5-gene in depression and treatment response—An association study in the Sequenced Treatment Alternatives to Relieve Depression (STAR*D) Cohort. Biol. Psychiatry.

[89] Sarginson J.E., Lazzeroni L.C., Ryan H.S., Schatzberg A.F., Murphy G.M. (2010). FKBP5 polymorphisms and antidepressant response in geriatric depression. Ame. J. Med. Genet. Part B Neuropsychiatr. Genet..

[90] Gassen N.C., Fries G.R., Zannas A.S., Hartmann J., Zschocke J., Hafner K., Carrillo-Roa T., Steinbacher J., Preissinger S.N., Hoeijmakers L. (2015). Chaperoning epigenetics: FKBP51 decreases the activity of DNMT1 and mediates epigenetic effects of the antidepressant paroxetine. Sci. Signal..

[91] Koch C.E., Leinweber B., Drengberg B.C., Blaum C., Oster H. (2017). Interaction between circadian rhythms and stress. Neurobiol. Stress.

[92] Pilz L.K., Carissimi A., Oliveira M.A.B., Francisco A.P., Fabris R.C., Medeiros M.S., Scop M., Frey B.N., Adan A., Hidalgo M.P. (2018). Rhythmicity of Mood Symptoms in Individuals at Risk for Psychiatric Disorders. Sci. Rep..

[93] Debono M., Ghobadi C., Rostami-Hodjegan A., Huatan H., Campbell M.J., Newell-Price J., Darzy K., Merke D.P., Arlt W., Ross R.J. (2009). Modified-release hydrocortisone to provide circadian cortisol profiles. J. Clin. Endocrinol. Metab..

[94] Chabot C.C., Taylor D.H. (1992). Daily rhythmicity of the rat acoustic startle response. Physiol. Behav..

[95] Dickmeis T. (2009). Glucocorticoids and the circadian clock. J. Endocrinol..

[96] Woodruff E.R., Chun L.E., Hinds L.R., Spencer R.L. (2016). Diurnal Corticosterone Presence and Phase Modulate Clock Gene Expression in the Male Rat Prefrontal Cortex. Endocrinology.

[97] Guillaumond F., Dardente H., Giguere V., Cermakian N. (2005). Differential control of Bmal1 circadian transcription by REV-ERB and ROR nuclear receptors. J. Biol. Rhythms.

[98] Zheng B., Larkin D.W., Albrecht U., Sun Z.S., Sage M., Eichele G., Lee C.C., Bradley A. (1999). The mPer2 gene encodes a functional component of the mammalian circadian clock. Nature.

[99] Bunger M.K., Wilsbacher L.D., Moran S.M., Clendenin C., Radcliffe L.A., Hogenesch J.B., Simon M.C., Takahashi J.S., Bradfield C.A. (2000). Mop3 is an essential component of the master circadian pacemaker in mammals. Cell.

[100] Zheng B., Albrecht U., Kaasik K., Sage M., Lu W., Vaishnav S., Li Q., Sun Z.S., Eichele G., Bradley A. (2001). Nonredundant roles of the mPer1 and mPer2 genes in the mammalian circadian clock. Cell.

[101] Scharf S.H., Liebl C., Binder E.B., Schmidt M.V., Müller M.B. (2011). Expression and Regulation of the Fkbp5 Gene in the Adult Mouse Brain. PLoS ONE.

[102] Blair L.J., Nordhues B.A., Hill S.E., Scaglione K.M., O’Leary J.C., Fontaine S.N., Breydo L., Zhang B., Li P., Wang L., Cotman C. (2013). Accelerated neurodegeneration through chaperone-mediated oligomerization of tau. J. Clin. Investig..

[103] Sabbagh J.J., O‘Leary J.C., Blair L.J., Klengel T., Nordhues B.A., Fontaine S.N., Binder E.B., Dickey C.A. (2014). Age-associated epigenetic upregulation of the FKBP5 gene selectively impairs stress resiliency. PLoS ONE.

[104] Myers B.L., Badia P. (1995). Changes in circadian rhythms and sleep quality with aging: Mechanisms and interventions. Neurosci. Biobehav. Rev..

[105] Albu S., Romanowski C.P., Letizia Curzi M., Jakubcakova V., Flachskamm C., Gassen N.C., Hartmann J., Schmidt M.V., Schmidt U., Rein T. (2014). Deficiency of FK506-binding protein (FKBP) 51 alters sleep architecture and recovery sleep responses to stress in mice. J. Sleep Res..

[106] Yuno A., Lee M.J., Lee S., Tomita Y., Rekhtman D., Moore B., Trepel J.B. (2018). Clinical Evaluation and Biomarker Profiling of Hsp90 Inhibitors. Methods Mol. Biol..

[107] Samuni Y., Ishii H., Hyodo F., Samuni U., Krishna M.C., Goldstein S., Mitchell J.B. (2010). Reactive oxygen species mediate hepatotoxicity induced by the Hsp90 inhibitor geldanamycin and its analogs. Free Radic. Biol. Med..

[108] Amin K., Ip C., Jimenez L., Tyson C., Behrsing H. (2005). In vitro detection of differential and cell-specific hepatobiliary toxicity induced by geldanamycin and 17-allylaminogeldanamycin using dog liver slices. Toxicol. Sci. Off. J. Soc. Toxicol..

[109] Rajan A., Kelly R.J., Trepel J.B., Kim Y.S., Alarcon S.V., Kummar S., Gutierrez M., Crandon S., Zein W.M., Jain L. (2011). A phase I study of PF-04929113 (SNX-5422), an orally bioavailable heat shock protein 90 inhibitor, in patients with refractory solid tumor malignancies and lymphomas. Clin. Cancer Res..

[110] Xie Q., Wondergem R., Shen Y., Cavey G., Ke J., Thompson R., Bradley R., Daugherty-Holtrop J., Xu Y., Chen E. (2011). Benzoquinone ansamycin 17AAG binds to mitochondrial voltage-dependent anion channel and inhibits cell invasion. Proc. Natl. Acad. Sci. USA.

[111] Gadelle D., Bocs C., Graille M., Forterre P. (2005). Inhibition of archaeal growth and DNA topoisomerase VI activities by the Hsp90 inhibitor radicicol. Nucleic Acids Res..

[112] Kijima T., Prince T.L., Tigue M.L., Yim K.H., Schwartz H., Beebe K., Lee S., Budzynski M.A., Williams H., Trepel J.B. (2018). HSP90 inhibitors disrupt a transient HSP90-HSF1 interaction and identify a noncanonical model of HSP90-mediated HSF1 regulation. Sci. Rep..

[113] Gaali S., Kirschner A., Cuboni S., Hartmann J., Kozany C., Balsevich G., Namendorf C., Fernandez-Vizarra P., Sippel C., Zannas A.S. (2015). Selective inhibitors of the FK506-binding protein 51 by induced fit. Nat. Chem. Biol..

[114] Zheng D., Sabbagh J.J., Blair L.J., Darling A.L., Wen X., Dickey C.A. (2016). MicroRNA-511 Binds to FKBP5 mRNA, Which Encodes a Chaperone Protein, and Regulates Neuronal Differentiation. J. Biol. Chem..

[115] Guy N.C., Garcia Y.A., Cox M.B. (2015). Therapeutic Targeting of the FKBP52 Co-Chaperone in Steroid Hormone Receptor-Regulated Physiology and Disease. Curr. Mol. Pharmacol..

[116] Cheung-Flynn J., Prapapanich V., Cox M.B., Riggs D.L., Suarez-Quian C., Smith D.F. (2005). Physiological role for the cochaperone FKBP52 in androgen receptor signaling. Mol. Endocrinol..

